# Influence of the Circadian Timing System on Tacrolimus Pharmacokinetics and Pharmacodynamics After Kidney Transplantation

**DOI:** 10.3389/fphar.2021.636048

**Published:** 2021-03-17

**Authors:** Pere Fontova, Helena Colom, Raül Rigo-Bonnin, Lisanne N. van Merendonk, Anna Vidal-Alabró, Nuria Montero, Edoardo Melilli, Maria Meneghini, Anna Manonelles, Josep M. Cruzado, Juan Torras, Josep Maria Grinyó, Oriol Bestard, Nuria Lloberas

**Affiliations:** ^1^ Nephrology Department, Bellvitge University Hospital, Barcelona, Spain; ^2^ Nephrology and Transplantation, Institut d'Investigació Biomédica de Bellvitge, Barcelona, Spain; ^3^ Department of Clinical Sciences, University of Barcelona, Barcelona, Spain; ^4^ Biopharmaceutics and Pharmacokinetics Unit, Department of Pharmacy and Pharmaceutical Technology, School of Pharmacy, University of Barcelona, Barcelona, Spain; ^5^ Biochemistry Department, Bellvitge University Hospital, Universitari de Bellvitge, Institut d'Investigació Biomédica de Bellvitge, Barcelona, Spain

**Keywords:** tacrolimus, pharmacodynamic, pharmacokinetics, circadian rhythm, kidney transplantation, immunosuppression

## Abstract

**Introduction:** Tacrolimus is the backbone immunosuppressant after solid organ transplantation. Tacrolimus has a narrow therapeutic window with large intra- and inter-patient pharmacokinetic variability leading to frequent over- and under-immunosuppression. While routine therapeutic drug monitoring (TDM) remains the standard of care, tacrolimus pharmacokinetic variability may be influenced by circadian rhythms. Our aim was to analyze tacrolimus pharmacokinetic/pharmacodynamic profiles on circadian rhythms comparing morning and night doses of a twice-daily tacrolimus formulation.

**Methods:** This is a post-hoc analysis from a clinical trial to study the area under curve (AUC) and the area under effect (AUE) profiles of calcineurin inhibition after tacrolimus administration in twenty-five renal transplant patients. Over a period of 24 h, an intensive sampling (0, 0.5, 1, 1.5, 2, 3, 4, 6, 8, 12, 12.5, 13, 13.5, 14, 15, 20, and 24 h) was carried out. Whole blood and intracellular tacrolimus concentrations and calcineurin activity were measured by UHPLC-MS/MS.

**Results:** Whole blood and intracellular AUC_12–24 h_ and C_max_ achieved after tacrolimus night dose was significantly lower than after morning dose administration (AUC_0–12 h_) (*p* < 0.001 for both compartments). AUE_0–12 h_ and AUE_12–24 h_ were not statistically different after morning and night doses. Total tacrolimus daily exposure (AUC_0–24 h_), in whole blood and intracellular compartments, was over-estimated when assessed by doubling the morning AUC_0–12 h_ data.

**Conclusion:** The lower whole blood and intracellular tacrolimus concentrations after night dose might be influenced by a distinct circadian clock. This significantly lower tacrolimus exposure after night dose was not translated into a significant reduction of the pharmacodynamic effect. Our study may provide conceptual bases for better understanding the TDM of twice-daily tacrolimus formulation.

## Introduction

Tacrolimus (Tac) is the most commonly used immunosuppressor after solid organ transplantation. After oral Tac administration, there is a large variability in the rate of absorption and bioavailability (Staatz and Tett, 2004; Hesselink et al., 2005). Therapeutic Tac doses are adjusted by monitoring the morning whole blood trough concentrations (C_trough_), even though some controversies remain regarding the relationship between C_trough_ and clinical outcomes. The area under concentration-time curve (AUC) is the most accurate assessment of overall Tac exposure although it is difficult to implement in clinical daily practice (Wallemacq et al., 2009). Therefore, routine therapeutic drug monitoring (TDM) of morning trough concentrations remains as the standard of care. Data of correlations between C_trough_ and AUC still remains a matter of discussion (Brunet et al., 2019). Marquet et al. (2018) reported that for twice-daily Tac formulation, the AUC_0–12 h_ correlated better with C_12_ than C_0_. A poor correlation between Tac dosage and trough levels exists, thus further research of factors influencing Tac exposure is strongly recommended (Hesselink et al., 2005; Wallemacq et al., 2009).

Immunosuppressive drugs have pharmacokinetic (PK) characteristics that may be influenced by circadian rhythms. These biological rhythms have been singled out as one of the causes of intra- and inter-patient variability (Baraldo and Furlanut, 2006). Several studies have shown the influence of circadian rhythms in gastric pH, gastric emptying time, gastrointestinal transit time, cytochrome P450 (CYP) activity in the liver and renal function, among others (Labrecque and Bélanger, 1991; Baraldo and Furlanut, 2006). Therefore, circadian rhythms affect processes of absorption, distribution, metabolism, and elimination of drugs and ultimately drug exposure and efficacy (Ohdo, 2007). In this context, differences in the P-glycoprotein (Pgp, encoded by *ABCB1* gene) efflux pump or in the CYP3A activity, could also affect morning and night Tac exposures (Hoffmeyer et al., 2000; Baraldo and Furlanut, 2006; Andreu et al., 2017).

In recent years, several studies have investigated the impact of circadian rhythms in twice-daily Tac formulation but whether there is a constant effect of Tac throughout a 24 h period remains to be concluded. Prado-Velasco et al. (2020) developed the first PK model-based study that supports the relationship between Tac concentration patterns and the circadian modulation of clearance and absorption suggesting that Tac intra-patient variability may be partially explained by circadian rhythms in Tac absorption and metabolism. However, most studies on twice-daily Tac formulation analyzed a low number of transplant patients, employed different Tac determination methodologies and fasting conditions (Min et al., 1996; Tada et al., 2003; Iwahori et al., 2005; Park et al., 2007; Satoh et al., 2008; Gustavsen et al., 2020). Notably, these studies have only evaluated Tac exposure after the morning dose during the 12 h intervals between the two daily doses (AUC_0–12 h_), but differences between the two AUCs (AUC_0–12 h_ and AUC_12–24 h_) still need to be studied more comprehensively (Min et al., 1996; Tada et al., 2003; Iwahori et al., 2005; Park et al., 2007; Gustavsen et al., 2020). Despite various reports analyzing the PK differences between day- and night-time of twice-daily Tac administration, results continue to be controversial. Furthermore, the effect of circadian rhythms on intracellular Tac concentrations and, ultimately, on different calcineurin (CN) activity, has not been fully investigated yet. The intracellular Tac concentrations on its target site of action could better reflect Tac exposure and it has been correlated with clinical outcomes (Capron et al., 2012; Han et al., 2016; Francke et al., 2020).

Our aim was to investigate whether the influence of circadian timing system on whole blood following Tac administration of twice-daily Tac formulations have an impact on intracellular lymphocyte Tac concentrations and on its pharmacodynamics (PD) (measured as CN activity inhibition). For this purpose, PK/PD analysis comparing morning and night Tac AUCs in renal transplant patients was performed.

## Methods

### Study Design

A *post-hoc* analysis of a prospective, non-randomized clinical trial was carried out at the Kidney Transplant Unit of Bellvitge University Hospital (clinicalTrials.gov NCT02961608) (Fontova et al., 2021). This clinical trial was conducted in accordance with the Declaration of Helsinki and with the local ethics committee. The study involved 25 adult recipients who received a kidney transplant at least 6 months before the inclusion. Main exclusion criteria for the PK/PD analysis included patients with severe gastrointestinal disorders or current infections and patients receiving concomitant drugs interacting with CYP3A enzymes. Recipients using twice-daily Tac formulation (Prograf® or Adoport®), with C_trough_ between 5 and 10 ng/ml and who signed informed consent were recruited in this study. No changes of Tac doses at least for 2 weeks before the PK/PD analysis was mandatory. During the period of 24 h, PK and PD analysis was conducted by an intensive sampling on the following time-points: Pre-dose and at 0.5, 1, 1.5, 2, 3, 4, 6, 8, and 12 following morning Tac dose and 12, 12.5, 13, 13.5, 14, 15, 20, and 24 h following night Tac dose. The night sampling was reduced according to the nursery blood draw logistics. Tac doses were carried out at least 1 h before and 2 h after meals, every 12 h (at 8:00 am and 08:00 pm) and all patients received the same Mediterranean diet (breakfast: 9:30 am, lunch: 2:00 pm, snack: 5:00 pm, dinner: 09:00 pm).

### Tacrolimus Measurement and Pharmacokinetic Data Analysis

The measurement of Tac concentrations in whole blood and intracellular in peripheral blood mononuclear cells (PBMCs) was performed using ultra-high-performance liquid chromatography coupled with tandem mass-spectrometry (UHPLC-MS/MS; Acquity®-TQD® mass spectrometer) using previously validated methods by our group (Rigo-Bonnin et al., 2015; van Merendonk et al., 2020). For intracellular Tac measurement, PBMCs isolation from whole blood was carried out using Ficoll density gradient. Thereafter, these PBMCs were lysed with a hypotonic lysis buffer. All Tac determinations showed concentrations higher than the limit of quantification either in whole blood or in intracellular compartments (0.65 and 0.126 ng/ml, respectively).

A non-compartmental PK analysis was carried out to estimate the most relevant exposure parameters from the individual concentration-time profiles obtained at steady-state conditions. Both the whole blood and intracellular concentration-time profiles were analyzed by using Phoenix-WinNonlin 64 v8.2. The parameters calculated were: trough concentration values (C_trough_), denoted as C_0_, trough concentration before morning dose; C_12_, trough concentration before night dose, C_24_, trough concentration 12 h after night dose; C_max_, peak concentration after each Tac dose; T_max_, time to reach C_max_; AUCs, areas under the concentration-time curves from 0 to 12 h time intervals after each morning (AUC_0–12 h_) and night (AUC_12–24 h_) doses calculated by the trapezoidal rule; Peak-trough fluctuation index (PTF) calculated as % PTF = 100 × [(C_max_ − C_trough_)/C_average_] where C_average_, was estimated from the ratios AUC_0–12_/τ or AUC_12–24_/τ where *τ* = 12 h; Swing fluctuation index (SFI) estimated as % SFI = 100 × [(C_max_ − C_trough_)/C_trough_]. Oral clearance values (CL/F) were also estimated from the ratios Dose/AUC.

### Calcineurin Activity Measurement and Pharmacodynamic Analysis

The PD effect of Tac was based on measurements of CN activity in PBMCs using a method previously validated by our group (Fontova et al., 2019). Briefly, once PBMCs were isolated and lysed with the hypotonic buffer used for intracellular Tac determination, the lysate was incubated for 15 min at 30°C with an exogenous phosphorylated peptide (RIIp). The calcium-dependent CN activity dephosphorylates RIIp, and after solid-phase extraction, dephosphorylated peptide (RII) and its corresponding internal-standard (RII-IS, isotope-labelled RII) were determined by UHPLC-MS/MS. All the extractions showed RII levels higher than the limit of quantification (0.04 µM).

Distinct PD parameters were calculated from the CN activity-time profiles at steady-state conditions in PBMCs by using Phoenix-WinNonlin 64 v8.2. The parameters calculated were: trough CN activity (I_trough_) denoted as, I_0_, trough CN activity before morning dose; I_12_, trough CN activity before night dose; I_24_, trough CN activity 12 h after night dose; I_min_, minimum inhibition of CN activity, I_nadir_, maximum CN inhibition; T_nadir_, time to achieve I_nadir_; AUEs, areas under the effect-time profiles from 0 to 12 h time intervals after morning (AUE_0–12 h_) and night (AUE_12–24 h_) doses estimated using the trapezoidal rule. The AUEs were evaluated from the percentage of inhibition curves considering either the I_min_ and I_nadir_ from the 24 h time interval period as baseline following the Eqs. 1, 2 respectively:

$$\%\;Inhibition_{Imin}\;=\left[\frac{\left(I_{min}-I_{x}\right)}{I_{min}}\right]*100$$ (1)

$$\%\;Inhibition_{Inadir}=\left[\frac{\left(Ix-I_{nadir}\right)}{I_{nadir}}\right]*100$$ (2)

where I_x_ was the CN activity at each experimental time.

### Genotyping

Genomic DNA was extracted from a peripheral whole-blood sample using Maxwell RSC® (Promega Corporation, Sydney, Australia) and was stored at −80°C. Genotyping of the CYP3A5*3 G > A (rs776746), CYP3A4*22 C > T (rs35599367) and *ABCB1 3435C > T* (rs1045642) polymorphisms (SNPs) was carried out using TaqMan SNP Genotyping Assay (Applied Biosystems, Foster City, CA, United States) in 384-well plates that included positive and negative controls. Real-time PCRs were carried out on the 7900HT Fast Real-time PCR System, Applied Biosystems (Thermo Fisher Scientific, Waltham, MA, United States), following standard recommendations. Briefly, 0.5 µl of each probe was mixed with 5 µl iTaq Universal Probes Supermix, 1 µl genomic DNA (10–20 ng/μl) and 3.5 µl of DNAse free water. The Real-time conditions were heat to 50°C for 2°min and 95°C for 10 min in the thermal cycler. This was then followed by 40 cycles of denaturization at 95°C for 15°s and annealing/extending at 60°C for 1 min. Samples were genotyped in CCiT-UB (Centres Científics i Tecnològics) at University of Barcelona, Campus Bellvitge.

### Statistical Analysis

Continuous variables derived from intracellular and whole blood Tac concentration-time and CN activity-time profiles were expressed as geometric mean [95% geometric mean interval confidence]. Tac exposure PK parameters and CN activity PD parameters estimated after the morning and night doses were compared by means of a two-sided paired t-test of natural log-transformed values. In contrast, Wilcoxon tests were performed for non-parametric variables such as T_max_ and T_nadir_. Correlations between the PD and PD parameters were evaluated by the parametric Pearson’s correlation test. Finally, demographic and non-continuous variables were described as median [interquartile range]. Statistical significance was set at *α* = 0.05. The statistical packages IBM SPSS v23 and Graphpad Prism 6.0 were used for the statistical analyses.

## Results

### Demographic Characteristics

Twenty-five renal transplant recipients from Bellvitge University Hospital were included in the clinical trial between 2016 and 2018. Patients received an immunosuppressive maintenance therapy consisting of twice-daily Tac with mycophenolate and corticosteroids. Three patients with asymmetric morning and night Tac doses were excluded from this PK/PD analysis. The demographic, *CYP3A* and *ABCB1* pharmacogenetics and clinical characteristics of the twenty-two patients are shown in Table 1.

**TABLE 1:** Baseline characteristics of the recipients included in the study (*n* = 22).

Variables	*N* = 22
Sex – Male/Female (%)	15/7 (68/32)
Age (years)	58.19 [48.24–69.08]
Time after transplantation (years)	2.12 [1.00–4.42]
Type of donor - deceased/Living (%)	19/3 (86/14)
Tacrolimus formulation – Prograf®/Adoport® (%)	9/13 (41/59)
Mycophenolate mofetil/sodium (%)	20/2 (91/9)
Other concomitant drugs (%)	
Prednisone	19 (76)
Omeprazole	20 (80)
Haematocrit (%)	39.05 [36.80–44.85]
Glomerular filtrate (ml/min)	48.00 [39.75–58.00]
Creatinine (µmol/L)	123.0 [104.0–169.0]
Albumin (g/L)	45.00 [42.75–47.00]
ALT (µkat/L)	0.26 [0.19–0.41]
GGT (µkat/L)	0.42 [0.32–0.73]
Genotype *CYP3A5* polymorphism (%)	
*1/*3	5 (23)
*3/*3	17 (77)
Genotype *CYP3A4* polymorphism (%)	
*1/*1	1 (5)
*1/*22	21 (95)
Genotype *ABCB1* polymorphism (%)	
*T/*T	6 (27)
*C-carriers	16 (73)

Numerical variables are expressed as median [interquartile range], whereas in parenthesis is represented the percentage for categorical data. CKD-EPI calculation was used for glomerular filtrate estimation. ALT, alanine aminotransferase; GGT, *γ*-glutamyl-transferase.

### Whole Blood Tacrolimus Pharmacokinetic Profile

**FIGURE 1:**
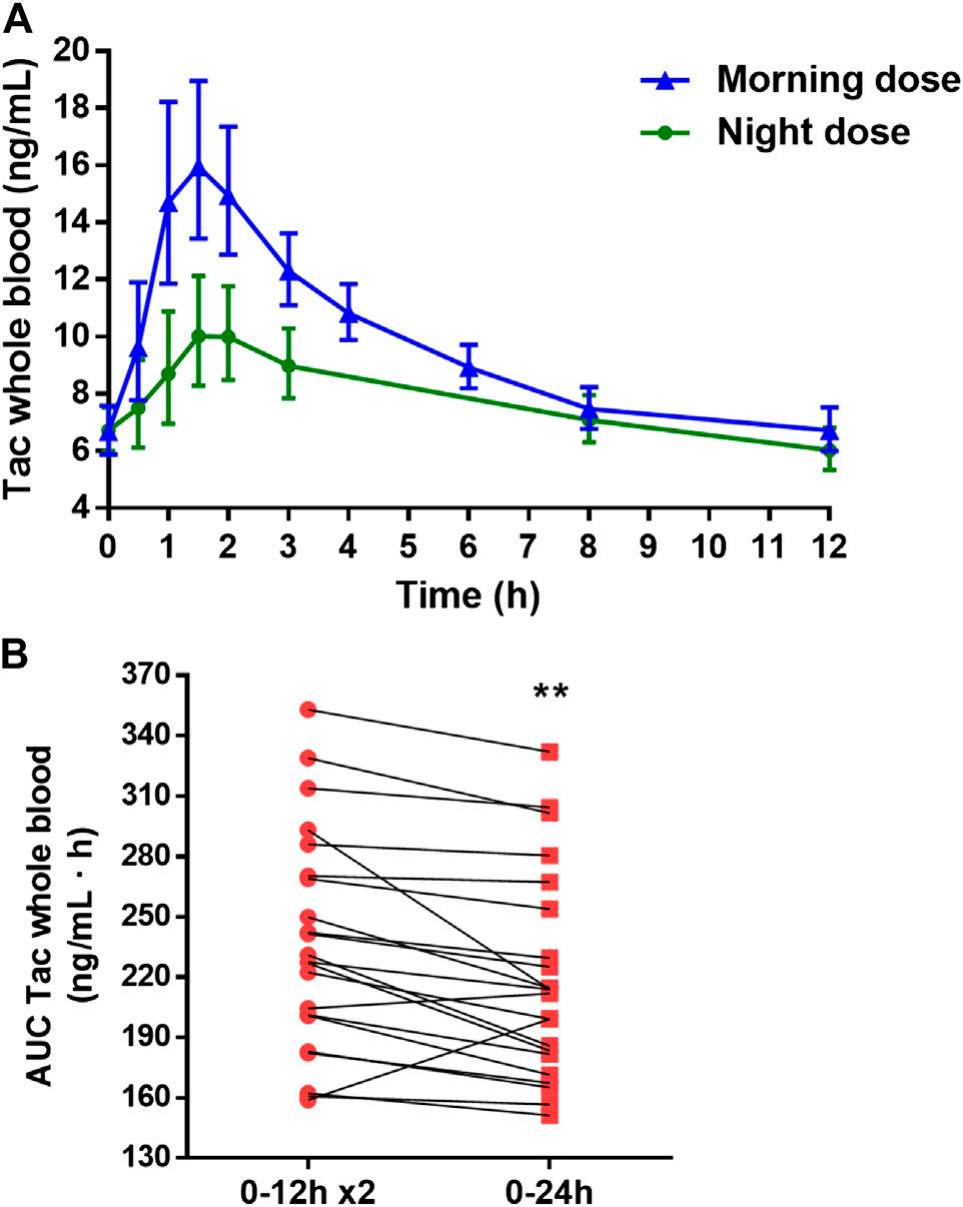
**(A)** Whole blood tacrolimus (Tac) concentration-time profiles along 12 h dose interval after the administration of morning and night dose of twice-daily Tac. **(B)**. Estimation of the total Tac daily exposure (AUC_0–24 h_) in whole blood (red square) by doubling the Tac exposure after the morning Tac dose (AUC_0–12_ x2) (red round). Each point joined by a line represented one patient. Paired *t*-test between both estimations was applied. **p < 0.01.

The observed mean Tac concentration-time profiles in whole blood after morning and night doses are shown in Figure 1A. The AUC, C_average_ and C_trough_ values were higher after the morning dose than the night doses (Table 2). CL/F was 25% higher after the morning dose with respect to the 12–24 h dosing interval. After the morning dose, a higher C_max_ was achieved than after the night dose. This was paired with a tendency to shorter T_max_ after the morning dose compared to the night dose, although it did not reach statistical significance (*p* = 0.182). Also, a higher fluctuation (PTF and SFI) was observed after morning dose as compared to the night dose, whereas C_trough_/AUC ratios were lower after the morning dose (Table 2).

**TABLE 2:** Comparison of tacrolimus (Tac) pharmacokinetic variables in whole blood and their corresponding correlations after morning Tac dose (0–12 h) and after night Tac dose (12–24 h) of twice-daily Tac formulation.

Variables	Morning dose (0–12 h)	Night dose (12–24 h)	*p*	
C_trough_ (ng/ml)	6.72 [6.00–7.52]	6.03 [5.34–6.82]	0.047^a^	
C_max_ (ng/ml)	18.20 [15.58–21.25]	11.12 [9.37–13.20]	<0.001^a^	
T_max_ (h)	1.52 [1.14–1.98]	1.87 [1.03–2.81]	0.182^b^	
AUC (ng·h/ml)	115.4 [104.2–127.9]	92.4 [81.5–104.8]	<0.001^a^	
C_trough_/AUC	0.058 [0.054–0.062]	0.065 [0.061–0.070]	0.014^a^	
PTF (%)	112.3 [92.3–136.6]	40.6 [22.4–73.4]	0.006^a^	
SFI (%)	161.6 [123.8–211.1]	46.5 [24.4–88.7]	0.003^a^	
C_average_ (ng/ml)	9.62 [8.68–10.66]	7.70 [6.79–8.73]	<0.001^a^	
CL/F (mg·L/ng·h)	15.74 [12.44–19.91]	19.66 [15.13–25.54]	<0.001^a^	
**Correlations** ^**c**^	**Morning dose (0–12 h)**		**Night dose (12–24 h)**	
	*R*	*P*	*R*	*p*
C_0_ vs AUC_0–12 h_	0.696	<0.001	—	—
C_12_ vs AUC_0–12 h_	0.810	<0.001	—	—
C_12_ vs AUC_12–24 h_	—	—	0.748	<0.001
C_24_ vs AUC_12–24 h_	—	—	0.856	<0.001
C_0_ vs C_max 0–12 h_	0.341	0.120	—	—
C_12_ vs C_max 12–24 h_	—	—	0.711	<0.001

^a^ Paired *t*-test.

^b^ Wilcoxon-test.

^c^ Pearson’s correlation test.

Data is represented as geometric mean [95% CI] unless T_max_, that is expressed as median [interquartile range]. C_trough_, trough concentration corresponding at time 12 h for morning dose and at time 24 h for night dose; C_max_, maximum peak Tac concentration; T_max_, time to reach C_max_; AUC, area under the concentration-time curve from each 12 h dose interval; PTF, peak-trough fluctuation index defined as [(C_max_ − C_trough_)/C_average_]; SFI, swing fluctuation index defined as [(C_max_ − C_trough_)/C_trough_]; C_average_, average Tac concentration from each 12 h dose interval; CL/F, clearance/bioavailability ratio; C_0_, morning pre-dose concentration at time 0 h; C_12_, night pre-dose concentration at time 12 h; C_24_, Tac concentration at time 24 h; AUC_0–12 h_, area under the concentration-time curve after the morning dose from 0 to 12 h; AUC_12–24 h_, area under the concentration-time curve after the night dose from 12 to 24 h.

Furthermore, a significant overestimation of the total daily Tac exposure was observed when the AUC_0–24 h_ was assessed by doubling the morning AUC_0–12_ (*p* = 0.002) (Figure 1B). In most patients, such overestimation was higher than 5% (16/22) and in some patients greater than 10% (8/22).

As also shown in Table 2, correlation between C_trough_ and C_max_ was only observed after the night dose. Strong correlations were also found between AUC_0–12_ and C_12_ and between AUC_12–24_ and C_24_ (*r* ∼ 0.8), although weaker correlations between AUC_0–12_ and C_0_ or AUC_12–24_ and C_12_ were also found (r ∼ 0.7). Scatter plots of these correlations are represented in Supplementary Figure S1.

The influence of genotypes on circadian rhythms was assessed. Frequencies observed in the present study were in accordance with reported allele frequencies in a Caucasian population and did not deviate from Hardy–Weinberg distribution. All patients were of Caucasian ethnicity. A three-way analysis of variance was applied for log-transformed normalized by dose AUC values with daytime (morning and night) and SNP (*CYPA3A4, CYP3A5 or ABCB1*) as fixed factors and patient as a random factor nested within SNP. Statistically significant differences were always found between daytimes (*p* < 0.001), *CYP3A5* SNP (*p* < 0.001), but not between *ABCB1* (*p* = 0.533) and *CYP3A4* SNPs (*p* = 0.324). Therefore, in this line, CYP3A5 1*/3* patients showed lower AUC/Dose than CYP3A5 3*/3* (*p* < 0.001) in both morning and night dose.

### Intracellular Tacrolimus Profile

**FIGURE 2:**
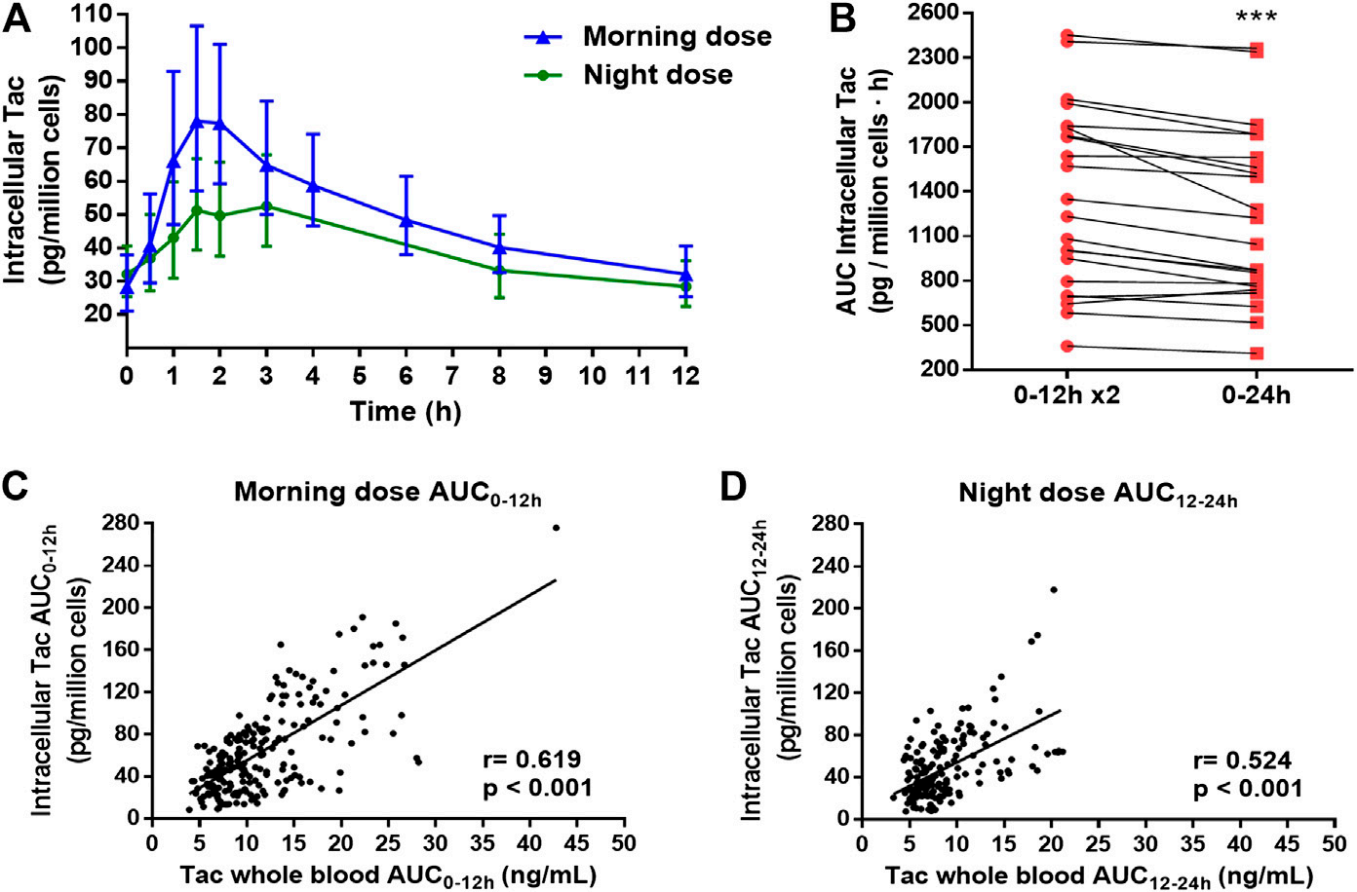
**(A)** Intracellular tacrolimus (Tac) concentration-time profiles along 12 h dose interval after the administration of morning and night dose of twice-daily Tac. **(B)**. Estimation of the total intracellular Tac daily exposure (AUC_0–24 h_) (red square) by doubling the Tac exposure after the morning Tac dose (AUC_0–12_ x2) (red round). Each point joined by a line represented one patient. Paired t-test between both estimations was applied. **p < 0.001. **(C,D)** Correlations between all whole blood Tac concentrations and intracellular Tac concentrations sampling after morning and night dose, respectively. Pearson test of Ln transformed data were performed for correlation analysis.

PK profiles of intracellular Tac measured in PBMCs were comparable to whole blood PK profiles (Figure 2A). Once again, Tac exposures given by AUCs, C_average_ and C_trough_ were higher in the morning when compared to the night dosing interval (Table 3). Similarly, to whole blood, after the morning Tac dose, a higher intracellular C_max_ was observed compared to the night dose. Meanwhile, no statistically significant differences were found between T_max_ values, nevertheless there was a tendency towards greater values at night with respect to the day-time dose (Table 3). This was in accordance with a more fluctuating profile (PTF and SFI values) after the morning dose in contrast to the night dose. No differences regarding, C_trough_/AUC ratios were found between both time settings (morning and night) although numerically higher values were observed after night dose (*p* = 0.085).

**TABLE 3:** Comparison of intracellular tacrolimus (Tac) pharmacokinetic variables and their corresponding correlations after morning Tac dose (0–12 h) and after night Tac dose (12–24 h) of twice-daily Tac formulation.

Variables	Morning dose (0–12 h)	Night dose (12–24 h)	*p*	
C_trough_ (pg/milion cells)	32.13 [25.41–40.62]	28.49 [22.42–36.21]	0.018^a^	
C_max_ (pg/million cells)	95.0 [72.0–125.3]	61.9 [47.6–80.5]	<0.001^a^	
T_max_ (h)	1.64 [1.14–2.11]	1.92 [1.46–2.89]	0.475^b^	
AUC (pg·h/million cells)	601.4 [478.1–756.5]	477.3 [373.7–609.7]	<0.001^a^	
C_trough_/AUC	0.053 [0.048–0.059]	0.059 [0.055–0.065]	0.085^a^	
PTF (%)	125.8 [105.6–149.9]	66.1 [51.9–84.2]	<0.001^a^	
SFI (%)	223.1 [176.5–297.0]	81.9 [59.6–112.4]	<0.001^a^	
C_average_ (pg/million cells)	50.12 [39.84–63.04]	39.78 [31.14–50,81]	<0.001^a^	
**Correlations** ^**c**^	**Morning dose (0–12 h)**		**Night dose (12–24 h)**	
	*R*	*p*	*R*	*p*
C_0_ vs AUC_0–12 h_	0.902^c^	<0.001	—	—
C_12_ vs AUC_0–12 h_	0.899^c^	<0.001	—	—
C_12_ vs AUC_12–24 h_	—	—	0.934^c^	<0.001
C_24_ vs AUC_12–24 h_	—	—	0.940^c^	<0.001
C_0_ vs C_max 0–12 h_	0.738^c^	<0.001	—	—
C_12_ vs C_max 12–24 h_	—	—	0.844^c^	<0.001

^a^ Paired *t*-test.

^b^ Wilcoxon-test.

^c^ Pearson’s correlation test.

Data is represented as geometric mean [95% CI] unless T_max_, that is expressed as median [interquartile range]. C_trough_, trough concentration corresponding at time 12 h for morning dose and at time 24 h for night dose; C_max_, maximum peak Tac concentration; T_max_, time to reach C_max_; AUC, area under the concentration-time curve from each 12 h dose interval; PTF, peak-trough fluctuation index defined as [(C_max_ − C_trough_)/C_average_]; SFI, swing fluctuation index defined as [(C_max_ − C_trough_)/C_trough_]; C_average_, average Tac concentration from each 12 h dose interval; C_0_, morning pre-dose concentration at time 0 h; C_12_, night pre-dose concentration at time 12 h; C_24_, Tac concentration at time 24 h; AUC_0–12 h_, area under the concentration-time curve after the morning dose from 0 to 12 h; AUC_12–24 h_, area under the concentration-time curve after the night dose from 12 to 24 h.

As in whole blood, the estimation of AUC_0–24 h_ by doubling the morning AUC_0–12 h_, was also overestimated in the intracellular compartment, although more patients displayed differences higher than 10% (13/22) (Figure 2B).

In contrast to whole blood, a significant correlation between C_trough_ and C_max_ after morning and night doses was observed in the intracellular compartment (Table 3). Stronger correlations between all possible combinations between trough concentrations and AUC values were observed compared to whole blood (*r* ∼ 0.9–0.95). The scatter plots representing these correlations are showed in Supplementary Figure S2. In addition, significant positive correlations were obtained between whole blood Tac concentrations and intracellular Tac concentrations either following morning dose or night dose (Figures 2C,D).

### Calcineurin Activity Profile

**FIGURE 3:**
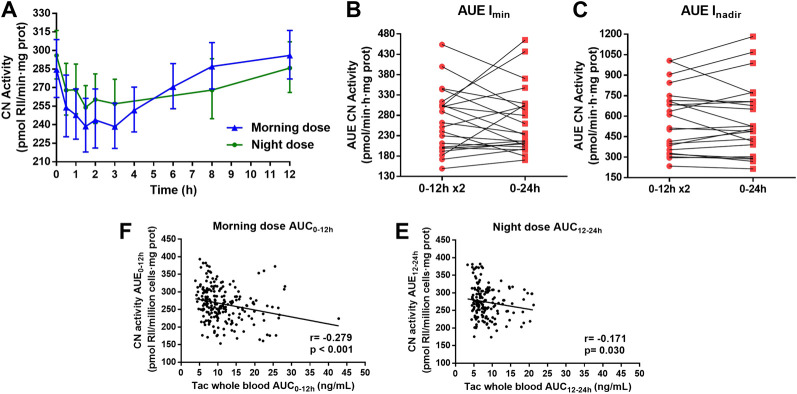
**(A)** Pharmacodynamic calcineurin (CN) activity-time profiles along 12 h dose interval after the administration of morning and night dose of twice-daily Tac. **(B)** Estimation of the total daily CN inhibition using *I*_min_ as baseline (AUE_0–24 h_ I_min_) (red square) by doubling the inhibition after the morning Tac dose (AUE_0–12_ I_min_ x2) (red round) and **(C)**, estimating the inhibition using I_nadir_ as baseline, AUE_0–24 h_ I_nadir_ (red square) and AUE_0–12 h_ I_nadir_ x2 (red round). Each point joined by a line represented one patient. Paired t-test between both estimations was applied. **(D,E)** Correlations between all whole blood Tac concentrations and CN activities sampling after morning and night dose, respectively. Pearson test of Ln transformed data were performed for correlation analysis.

The CN activity-time profiles in PBMCs after morning and night Tac doses are shown in Figure 3A. Similar to whole blood and intracellular PK profiles, there were clear differences in CN activity profiles regarding day and night times. Indeed, more fluctuation occurred after the morning dose. The morning dose also showed a statistically significant lower I_nadir_, indicating higher CN inhibition, than the night dose (Table 4). Although a significantly higher I_trough_ was observed after the night dose with respect to morning dose, the ratio of I_trough_/I_nadir_ was still significantly higher after the morning dose. Moreover, no differences in T_nadir_ were found between both Tac doses (Table 4). The morning dose showed a comparable AUE I_min_ and AUE I_nadir_ to the night dose when compared to AUC in whole blood or intracellular PK (Table 4).

**TABLE 4:** Comparison of pharmacodynamic variables measured as calcineurin (CN) activity and their corresponding correlations after morning tacrolimus dose (0–12 h) and after night Tac dose (12–24 h) of twice-daily tacrolimus formulation.

Variables	Morning dose (0–12 h)	Night dose (12–24 h)	*p*	
I_trough_ (pmol RII/min·mg prot)	296.0 [277.1–316.3]	285.8 [266.1–307.0]	0.040^a^	
I_nadir_ (pmol RII/min·mg prot)	220.1 [204.3–237.2]	238.7 [221.7–257.0]	0.002^a^	
T_nadir_ (h)	2.16 [1.25–3.49]	2.76 [1.47–4.26]	0.656^b^	
I_trough_/I_nadir_	1.29 [1.25–1.34]	1.20 [1.15–1.24]	0.002^a^	
AUE I_min_ (pmol RII·h/min·mg prot)	127.7 [112.3–145.2]	120.1 [99.1–145.6]	0.572^a^	
AUE I_nadir_ (pmol RII·h/min·mg prot)	262.4 [216.2–318.4]	245.9 [190.6–317.3]	0.449^a^	
**Correlations** ^**c**^	**Morning dose (0–12 h)**		**Night dose (12–24 h)**	
	*R*	*p*	*R*	*P*
I_0_ vs AUE_0–12 h_ I_min_	−0.034	0.881	—	—
I_12_ vs AUE_0–12 h_ I_min_	0.115	0.609	—	—
I_12_ vs AUE_12–24 h_ I_min_	—	—	−0.001	0.968
I_24_ vs AUE_12–24 h_ I_min_	—	—	−0.255	0.252
I_0_ vs AUE_0–12 h_ I_nadir_	−0.134	0.552	-	-
I_12_ vs AUE_0–12 h_ I_nadir_	0.125	0.579	-	-
I_12_ vs AUE_12–24 h_ I_nadir_	—	—	0.158	0.491
I_24_ vs AUE_12–24 h_ I_nadir_	—	—	0.252	0.257
I_0_ vs I_nadir 0–12 h_	0.899	<0.001		
I_12_ vs I_nadir 12–24 h_			0.818	<0.001

^a^ Paired *t*-test.

^b^ Wilcoxon test.

^c^ Pearson’s correlation test.

Data is represented as geometric mean [95% CI] unless T_nadir_ that is expressed as median [interquartile range]. I_trough_, trough CN activity at time 12 h for morning dose and at time 24 h for night dose; I_nadir_, maximum inhibition of CN activity; T_nadir_, time to reach I_nadir_; AUE I_min_, area under the effect-time curve of CN inhibition from each 12 h dose interval using I_min_ as baseline; I_min_, minimum CN inhibition observed along 24 h interval; AUE I_nadir,_ area under the effect-time curve of CN inhibition from each 12 h dose interval using the I_nadir_ observed along 24 h interval as baseline; I_0_, morning pre-dose CN activity at time 0 h; C_12_, night pre-dose CN activity at time 12 h; C_24_, CN activity concentration at time 24 h; AUE_0–12 h_, area under the effect-time curve after the morning dose from 0 to 12 h; AUE_12–24 h_, area under the effect-time after the night dose from 12 to 24 h.

In contrast to whole and intracellular PK estimation, similar CN inhibition during a 24 h period (AUE_0–24_ I_min_ and AUE_0–24_ I_nadir_) was obtained when this was assessed by doubling the morning AUE_0–12 h_ (*p* = 0.972 and 0.826, respectively) (Figures 3B,C). Furthermore, concerning PD, high interpatient variability was observed in this estimation and few patients displayed differences lower than 10% in AUE_0–24_ I_min_ (7/22) and in AUE_0–24_ I_nadir_ (11/22).

No correlations between I_trough_ (I_0_, I_12_, I_24_) and AUEs were observed either in the morning nor in the night dose. However, in both Tac doses, a strong correlation was observed between I_0_ and I_nadir_ (*r* > 0.8) (Table 4). Scatter plots of these correlations are represented in Supplementary Figure S3. In addition, significant inverse weak correlation was observed between CN activities determinations and whole blood Tac concentrations both after morning dose and night dose (Figures 3D,E).

## Discussion

To the best of our knowledge, this is the first study that simultaneously evaluates the 24 h time variation in the pharmacokinetics at steady-state conditions of intracellular and whole blood Tac, after twice-daily administration. While the impact on circadian rhythms on whole blood Tac has previously been documented (Min et al., 1996; Tada et al., 2003; Iwahori et al., 2005; Park et al., 2007; Satoh et al., 2008; Gustavsen et al., 2020), data regarding intracellular Tac has yet to be reported. Here, we show the influence of circadian rhythms on intracellular PK after twice-daily Tac administration and their corresponding PD profiles in kidney transplant recipients. Notably, we also describe the differences in PK and PD profiles between the morning and night administration doses in a standard immunosuppressive regimen based on a twice-daily Tac formulation.

Our results show different whole blood PK profiles between the morning and the night Tac doses. Achieved exposures (AUC) following the night dose were approximately 25% lower than those achieved following the morning dose. Fluctuations of whole blood concentrations were also much lower during the 12–24 h dose intervals rather than after the morning dose. These results suggest 24 h variations in both the extent and rate of absorption due to physiological rhythms. Tac is a highly lipophilic drug with poor aqueous solubility. This is one of the factors contributing to its low and variable oral bioavailability, but also cytochrome P450 (CYP3A) mediated metabolism or multidrug-resistance associated protein-mediated efflux should be considered. Indeed, a day-dependent variability has been reported for *CYP3A* gene expression and mediated metabolism of other several drugs (Martin et al., 2003; Tomalik-Scharte et al., 2014). In this context, the lower Tac exposure after night dose could be due to an enhanced CYP3A enzymatic activity during night-time compared to day-time. This could lead to higher pre-systemic loss of Tac with lower fraction reaching the bloodstream and, in turn, to higher CL/F values at the 12–24 h dose interval with respect to the morning drug intake (19.66 and 15.74 mg·l/ng·h, respectively). In our study we found lower doses in patients who expressed *CYP3A^∗^3*/*^∗^3* SNP compared with expressors of *CYP3A^∗^1*/*^∗^3*. However, no differences were observed when AUC/Dose was compared for each individual SNP between morning and night dose. The study should be performed with a larger sample size to describe the impact of genotypes on circadian rhythms. Preclinical studies in different murine models (Murakami et al., 2008; Okyar et al., 2019) and in non-humans primates (Iwasaki et al., 2015) have also shown the influence of circadian variation on the expression and activity of the Tac extrusion transporter Pgp, in the intestine due to its regulation by *Clock* genes. A higher Pgp activity during the night period also may have contributed to the differences between morning and night Tac administrations observed. Tamura et al. (2003) reported a Tac permeability two times greater in the upper part of the intestine (jejunum) than in the ileum of rats. By contrast, Pgp activity was dominant in ileum compared to the jejunum. Considering Tac twice-daily as an immediate release formulation, major Tac uptake would be expected in the jejunum. At this point, circadian changes on CYP3A enzymatic activity would play a more important key role in the different exposures observed between morning and night administrations than Pgp extrusion. Our results are in line with those of Prado-Velasco et al. (2020) in the pediatric population describing the effect of circadian rhythms on CL.

We also found significantly lower fluctuation values in whole blood Tac concentrations following the night dose when compared to the morning dose (PTF, 40.6 and 112.3%; SFI, 46.5 and 161.6%, respectively). These results suggest a lower absorption rate during the night-time as it was described in the model of Prado-Velasco et al. (2020). This was also confirmed by a trend to larger T_max_ values at night with respect to the morning dose (1.87 and 1.52 h, respectively). Differences between C_trough_/AUC ratios were also observed. Lower values of this ratio were observed in the morning compared to the night dose interval, as should be expected when a faster absorption process takes place (0.058 and 0.065, morning and night respectively). Differences in the absorption rate could be explained by distinct fasting conditions before drug administration or reduced gastric emptying rate due to physiologically slower enterokinetics in the evening with respect to the day time. Indeed, food intake, especially after high-fat meals, diminished Tac levels and also slowed the absorption process as previously reported by Bekersky et al. (2001). Interestingly, Gustavsen et al. (2020), recently described that the daily circadian PK variations were largely affected by the fasting conditions at the time of drug intake showing lower C_max_ and AUC_0–12_ in non-fasting conditions after morning Tac dose. In this context, the different eating habits in relation to morning and evening doses should be considered as eating habits could influence Tac exposure. The significantly higher C_max_, C_max_/C_trough_, and AUC observed following morning Tac dose compared to night dose were in accordance with some previous reports (Min et al., 1996; Iwahori et al., 2005; Park et al., 2007; Gustavsen et al., 2020), although other studies did not find these PK differences (Tada et al., 2003; Satoh et al., 2008; Gustavsen et al., 2020). However, in the vast majority of these studies the morning Tac dose intake was undertaken after breakfast and not in fasting conditions, which differs to our study (Tada et al., 2003; Satoh et al., 2008).

To our knowledge, our study is the first showing the role of circadian rhythms on intracellular Tac PK. In this study, whole blood and intracellular exposures revealed pharmacokinetic profiles with a similar pattern. Both intracellular and whole blood concentrations were determined under steady-state conditions, and kinetic equilibrium between whole blood and PBMC (intracellular) compartment should have been achieved. Certainly, peak concentrations were observed at similar times for whole blood and intracellular Tac. As in whole blood, the morning dose resulted in a higher exposure than that of the night dose (601.4 and 477.3 pg·h/million cells, respectively). Intracellular peak concentrations were also higher at the 0–12 h interval than the 12–24 h interval (95.0 and 61.9 pg/million cells, respectively) resulting in higher fluctuation (PTF, 125.8 and 66.1%; SFI, 223.1 and 81.9%, respectively). Results obtained after the morning Tac dose from the first AUC_0–12 h_ were in accordance with previous studies (Lemaitre et al., 2015; Klaasen et al., 2018). The lower C_max_/C_trough_ ratio, especially after the morning dose, achieved intracellularly compared to whole blood suggests a restricted entrance of Tac molecules inside the cells. It is known that *ABCB1* polymorphisms affect the intracellular Tac exposure in PBMCs (Capron et al., 2010; Tron et al., 2020). However, no literature has been published to describe the role of circadian rhythms and Pgp on intracellular Tac exposure.

A specular PD/PK profile (whole blood or intracellular) was shown in our study after either the morning or night Tac dose. Thus far, the PD analysis measuring CN activity has only been studied following the morning dose of twice-daily Tac formulation, but no data has reported the PD effect after the night dose. Our data showed that despite a lower I_nadir_ following the morning Tac dose, this was not translated into higher CN activity inhibition during the first 12 h, thus showing comparable AUE_0–12 h_ and AUE_12–24 h_. This may be explained by the transient PD profile observed after morning Tac dose characterized by a rapid return to pre-dose levels once the I_nadir_ was reached, which differs to the more sustained inhibition after the I_nadir_, which was noticed following night dose. Previous studies also showed this rapid recovery of CN activity to pre-dose levels after the morning Tac dose (Koefoed-nielsen and Gesualdo, 2002; Koefoed-Nielsen et al., 2006; Iwasaki et al., 2018; Fontova et al., 2021). Other studies investigating lymphocyte activation have shown circadian rhythms displaying higher proinflammatory cytokine secretion during night time (Benedict et al., 2007; Fortier et al., 2011).

Until now, most studies analyzing the PK and the PD properties of twice-daily Tac formulation described the morning AUC_0–12 h_ as 50% of the total daily dose. The introduction of new once-daily Tac formulations was brought in to assess AUC_0–24 h_ even when it was compared with twice-daily Tac (Tsuchiya et al., 2013; Tremblay et al., 2017; Marquet et al., 2018). Despite the differences observed after night Tac dose in fasting conditions, some reports doubled the analysis of the first AUC_0–12 h_ to illustrate total daily dose exposure to compare once-and twice-daily Tac formulations (Iwasaki et al., 2018). Our results showed that by doubling the AUC_0–12 h_ after morning Tac dose, the AUC_0–24 h_ was overestimated either in whole blood or intracellular. High interpatient variability was observed in PD, which suggests that this estimation should be avoided. Therefore, the best approach to evaluate real AUC_0–24 h_ for twice-daily Tac is to measure both AUCs and not using extrapolations.

The TDM of Tac is based on measuring the whole blood morning C_trough_ levels (Wallemacq et al., 2009; Brunet et al., 2019). Even though measuring the morning C_0_ also showed good correlation with AUC_0–12 h_, it does not reflect the differences observed between morning and night AUC and C_max_. Intracellular results also reinforced these observations (Tron et al., 2020). Furthermore, our results showed that the I_0_, I_12_, and I_24_ did not correlate with their previous or posterior AUEs, suggesting that only measuring pre-dose CN activity is not sufficient to predict the total CN inhibition after twice-daily Tac dose.

In conclusion, our data proves that despite a clear impact of circadian rhythms on whole blood and intracellular Tac PK, ultimately this effect has a modest impact on Tac PD evaluated as the degree of CN inhibition. Our study may provide a conceptual basis for a better understanding of PD/PK Tac properties of twice-daily Tac formulation in recipients of renal transplantation.

## Data Availability

The raw data supporting the conclusions of this article will be made available by the authors, without undue reservation.

## Supplementary Material

FIGURE S1 | **Cortical hemodynamic results**. A and B. Correlation between whole blood pre-dose concentrations (C_0_ and C_12_) and their corresponding maximum peak-concentration (C_max_; C_max 0-12h_ and C_max 12-24h_). C and D. Correlation between whole blood trough concentrations (C_0_ and C_12_) and their corresponding posterior area under the curve (AUC; AUC_0-12h_ and AUC_12-24h_). E and F. Correlation between whole blood trough concentrations (C_12_ and C_24_) and their corresponding previous AUC (AUC_0-12h_ and AUC_12-24h_). For correlation analysis, Pearson test of Ln transformed data were used.

FIGURE S2 | **Cortical hemodynamic results**. A and B. Correlation between intracellular pre-dose concentrations (C_0_ and C_12_) and their corresponding maximum peak-concentration (C_max_; C_max 0-12h_ and C_max 12-24h_). C and D. Correlation between intracellular trough concentrations (C_0_ and C_12_) and their corresponding posterior area under the curve (AUC; AUC_0-12h_ and AUC_12-24h_). E and F. Correlation between intracellular trough concentrations (C_12_ and C_24_) and their corresponding previous AUC (AUC_0-12h_ and AUC_12-24h_). For correlation analysis, Pearson test of Ln transformed data were used.

FIGURE S3 | **Cortical hemodynamic results**. A and B. Correlation between trough CN activities (I_0_ and I_12_) and their corresponding posterior AUE I_min_ (AUE_0-12h_ I_min_ and AUE_12-24h_ I_min_). C and D. Correlation between trough CN activities (I_12_ and I_24_) and their corresponding previous AUE I_min_ (AUE_0-12h_ I_min_ and AUE_12-24h_ I_min_). E and F. Correlation between trough CN activities (I_0_ and I_12_) and their corresponding posterior AUE Inadir (AUE_0-12h_ I_nadir_ and AUE_12-24h_ I_nadir_). G and H. Correlation between trough CN activities (I_12_ and I_24_) and their corresponding previous AUE Inadir (AUE_0-12h_ I_nadir_ and AUE_12-24h_ I_nadir_). I and J. Correlation between pre-dose CN activities (I_0_ and I_12_) and their corresponding maximum peak-inhibition (I_nadir_; I_nadir 0-12h_ and I_nadir 12-24h_). For correlation analysis, Pearson test of Ln transformed data were used.

## Abbreviation

RRI, resource rdentification initiative.
