# Supplementary figures and images for: Influence of the Circadian Timing System on Tacrolimus Pharmacokinetics and Pharmacodynamics After Kidney Transplantation
Source: Front Pharmacol. 2021 Mar 17;12:636048. doi: 10.3389/fphar.2021.636048 (PMC8010682; doi:10.3389/fphar.2021.636048)

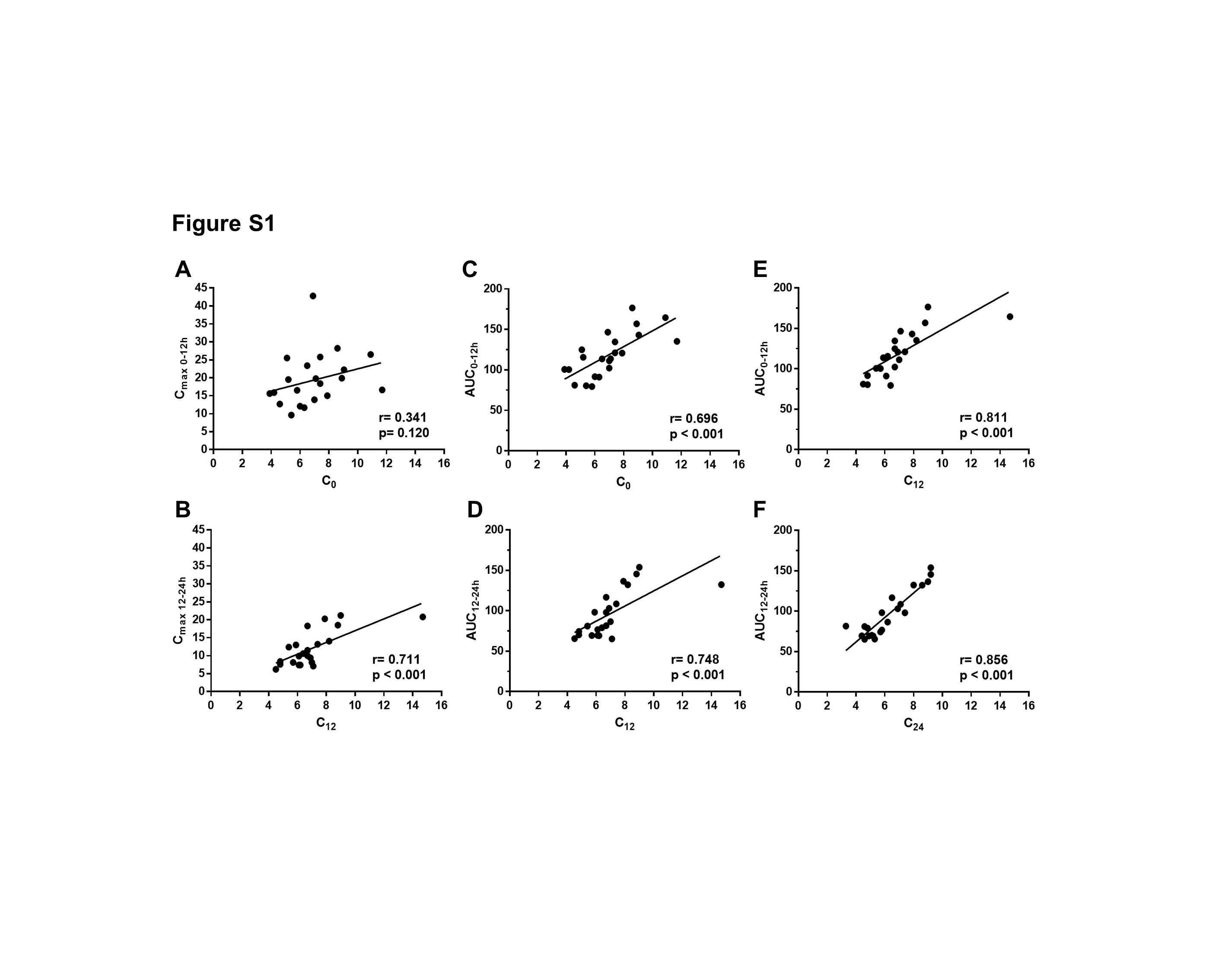

Supplement: Supplementary file 1 [file image1.jpeg]

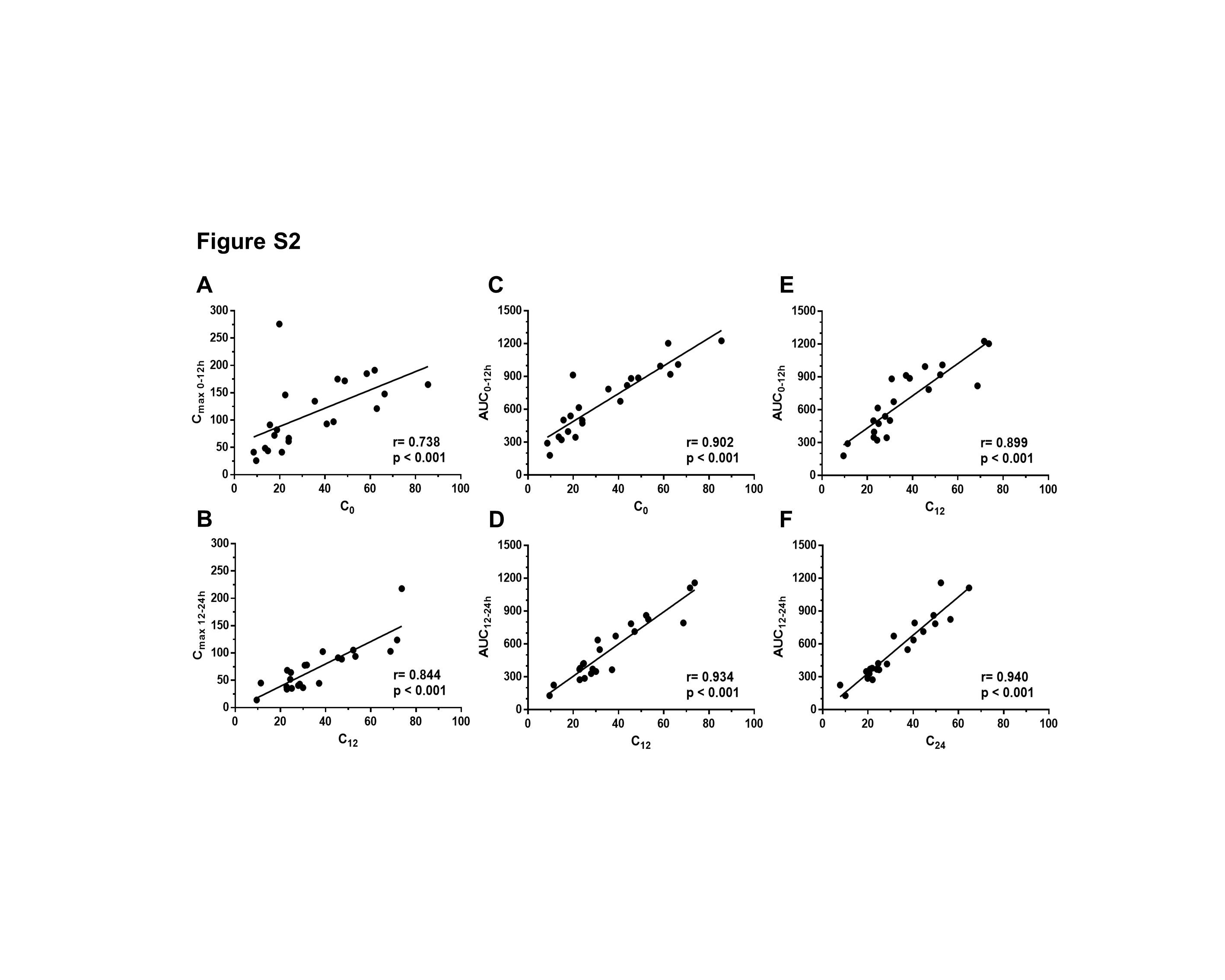

Supplement: Supplementary file 2 [file image2.jpeg]

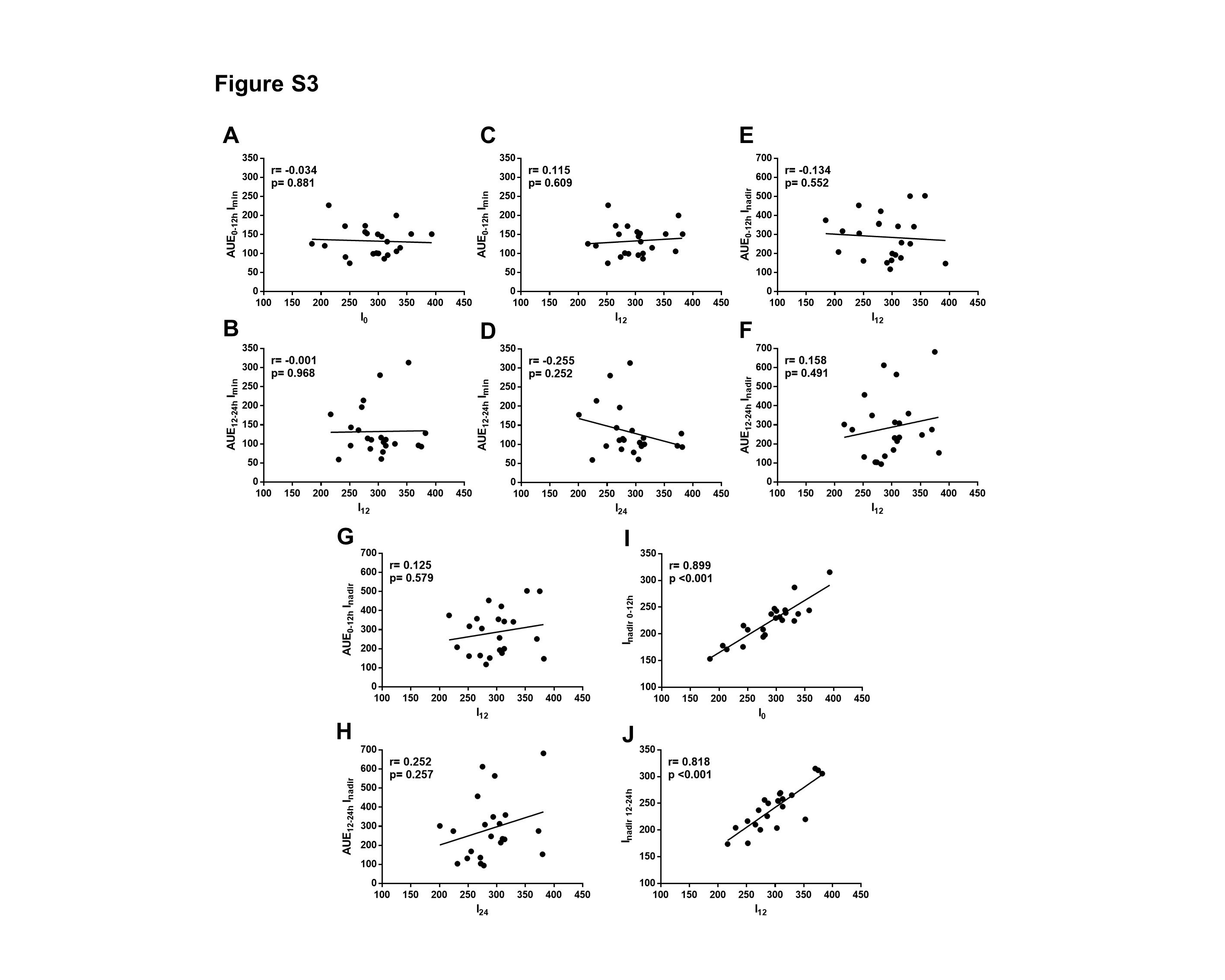

Supplement: Supplementary file 3 [file image3.jpeg]
